# Takotsubo cardiomyopathy with immune-mediated comorbidities: a case report

**DOI:** 10.1093/ehjcr/ytaf193

**Published:** 2025-04-17

**Authors:** Yuqing Chen, Joseph Cuthbert, Labib Khan, Katarzyna Malaczynska-Rajpold

**Affiliations:** School of Clinical Medicine, University of Cambridge, Addenbrooke's Hospital NHS Foundation Trust, Hills Rd, Cambridge CB2 0SP, UK; School of Clinical Medicine, University of Cambridge, Addenbrooke's Hospital NHS Foundation Trust, Hills Rd, Cambridge CB2 0SP, UK; School of Clinical Medicine, University of Cambridge, Addenbrooke's Hospital NHS Foundation Trust, Hills Rd, Cambridge CB2 0SP, UK; Lister Hospital, East and North Hertfordshire NHS Trust, Coreys Mill Lane, Stevenage SG1 4AB, UK; Royal Brompton Hospital, Guy’s and St Thomas’ NHS Foundation Trust, Sydney St, London SW3 6NP, UK

**Keywords:** Case report, Takotsubo cardiomyopathy, Broken heart syndrome, Autoimmune comorbidities

## Abstract

**Background:**

Takotsubo cardiomyopathy (TCM) is an under-recognized cardiovascular syndrome, leading to myocardial infarction and left ventricular systolic dysfunction, in the absence of detectable coronary artery lesion. The pathophysiologic mechanisms underlying the condition remain unclear. Whilst previously believed to be a result of circulating high levels of catecholamines due to preceding severe emotional or physical stress, giving it its colloquial name of ‘broken heart syndrome’, newer evidence suggesting accompanying systemic inflammatory activation hints at a mechanism of acute stress encounters serving as the trigger for an enhanced systemic and hence myocardial inflammation.

**Case summary:**

In this report, we present a 72-year-old female patient presenting to ED with sepsis, whose echocardiogram upon admission showed the entire mid and apical segments of the left ventricle as akinetic, with residual contractility only on the basal segments, suggestive of TCM as a likely diagnosis. This is on a backdrop of a complex history of autoimmune and chronic inflammatory comorbidities, including type 1 diabetes, asthma, sarcoidosis, primary biliary cholangitis, Sjogren’s syndrome, and type 3 cryoglobulinaemia.

**Discussion:**

In line with previous reports, this adds to the evidence base for the potential immune-mediated pathophysiology of TCM, highlighting inflammatory activation as causative as opposed to consequential to TCM, given its greater propensity for development of the disease in low-grade chronic inflammatory states. This identifies an understudied aspect of its aetiology, warranting further clinical and mechanistic investigation with implications for treatment and prophylaxis. Additionally, this highlights an important differential for acute myocardial infarction, in patients with underlying chronic inflammatory and autoimmune diseases.

**Learning points**

- Takotsubo cardiomyopathy (TCM) can occur in patients with autoimmune comorbidities, suggesting an immune-mediated pathophysiology.
- In patients with chronic inflammatory conditions, TCM may be triggered by acute stress, emphasizing the need for careful monitoring during such events.
- Recognition of TCM in patients with autoimmune diseases can aid in differential diagnosis and management.

## Introduction

Takotsubo cardiomyopathy (TCM) is an under-recognized cardiovascular syndrome, leading to myocardial infarction and left ventricular systolic dysfunction, in the absence of detectable coronary artery lesion.^1^ First reported since the 1990s, the name originates from the Japanese translation of ‘octopus trap’, to describe the shape of the heart during ventriculography, resembling a wide-based clay jar with a narrow neck for octopus fishing, due to its reversible apical ballooning in the early course of the disease.^2^

The pathophysiologic mechanisms underlying the condition remain unclear. The disease is often preceded by severe emotional or physical stress, giving it its colloquial name of ‘broken heart syndrome’, and hence a widely accepted theory is that circulating high levels of catecholamines result in myocardial microvascular alterations leading to negative inotropy and resultant left ventricular contractile dysfunction.^3^ With sepsis-induced myocardial depression showing reversible left ventricular dysfunction consistent with TCM, this theory aligns with the sequelae of acute cardiac sympathetic disruption with noradrenaline spill-over.^4^

Newer evidence shows accompaniment by systemic inflammatory activation, characterized by macrophage infiltration in the myocardium, increased pro-inflammatory monocyte subsets (CD14++ CD16−), and increased levels of circulating pro-inflammatory cytokines (IL-6, IL-8, and CXCL1).^5^ With some systemic changes persisting for over 5 months such as increased IL-6 and decreased intermediate monocytes, this demonstrates an evolvement into a low-grade, chronic inflammatory state, perhaps explaining the subset of patients lacking functional recovery. This case report adds to the growing evidence base of the association of TCM with pre-existing chronic inflammatory disorders. This apparent propensity for development of the disease in low-grade chronic inflammatory states warrants investigations into the pathophysiological mechanisms of underlying inflammatory processes.

## Summary figure

This is a video—please double click on this document or see the attached Supplementary figure in MP4 format.

**Figure:**
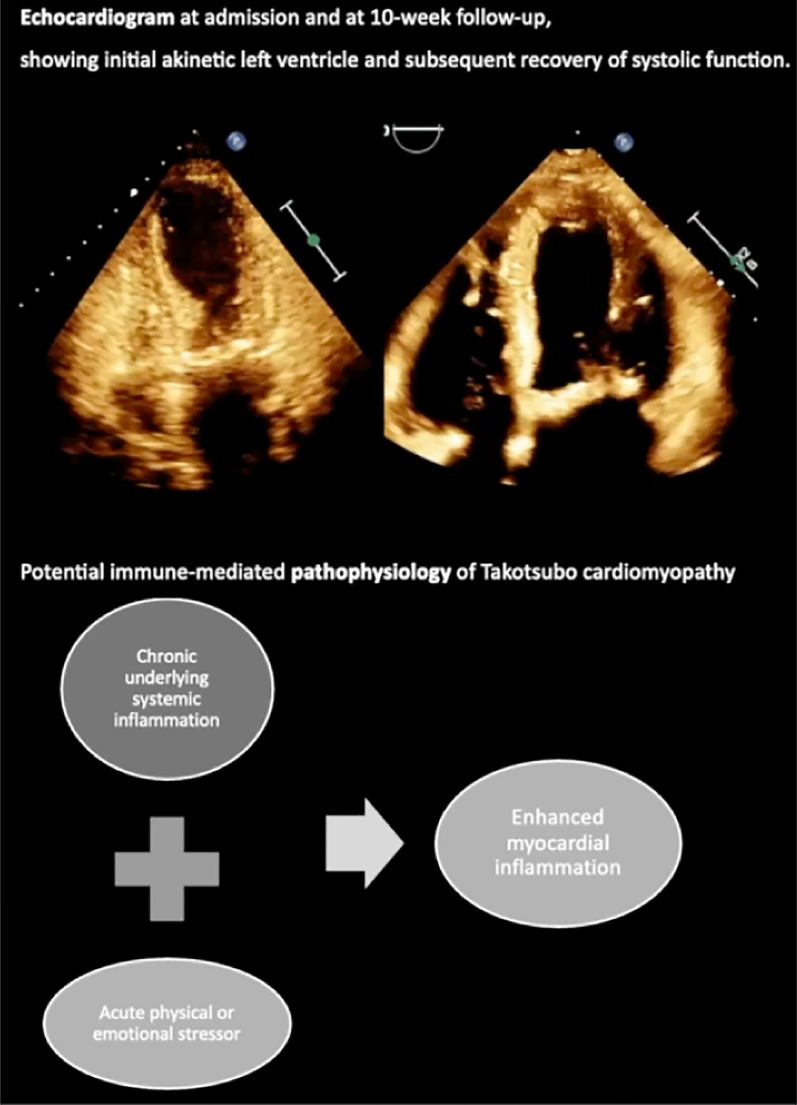


## Case presentation

A 72-year-old woman presented to the emergency department in the early morning with nausea and vomiting following a two-week course of doxycycline and clarithromycin for a lower respiratory tract infection. She was admitted for sepsis and underwent acute management, including fluid challenge and i.v. antibiotics as required. Her condition stabilized throughout the night and was transferred to the acute medical unit. The patient had a cardiac history, including severe mitral stenosis with complete heart block, managed with a permanent pacemaker. The physical examination included signs of heart failure including generalized oedema and signs of fluid overload, and a pan-systolic murmur of mitral regurgitation.

The echocardiogram at admission (Video 1) showed the entire mid and apical segments of the left ventricle (LV) as akinetic, with residual contractility only on the basal segments. Overall systolic function was severely impaired in the context of extensive regional wall motion abnormalities (WMAs) with an ejection fraction (EF) by Simpson’s biplane of 35%. LV diastology was unable to be assessed due to valvular disease and paced rhythm. Given this regional systolic dysfunction of the LV with coincident WMAs, this was suggestive of TCM and hence the patient was referred for further investigation by cardiac angiography. Subsequent angiogram (Video 2) showed mildly calcified coronaries, with the extent of coronary artery disease not justifying the LV impairment. There was also evidence of degenerative calcification of atrial valve and mitral valve, mild-to-moderate ostial atheroma of left anterior descending, diffuse disease of diagonal, moderately diffuse disease of left circumflex, and moderate 60% proximal disease of right coronary artery.

Her history included a multitude of pre-existing autoimmune and chronic inflammatory conditions, including type 1 diabetes mellitus, asthma, sarcoidosis, primary biliary cholangitis, Sjogren’s syndrome, and type 3 cryoglobulinaemia. Notably, the patient was known to have contracted rheumatic fever as a child, an autoimmune inflammatory response to streptococcal pharyngitis. Given post-infectious sequelae of acute rheumatic fever triggering valvular damage with preferential involvement of the mitral valve, this suggests a fitting autoimmune basis to the valvular heart disease. Her pre-morbid baseline state maintained independence in her basic activities of daily living, but requires assistance from social care and family support for complex needs. Together, the patient presents with an extensive history of comorbidities. Whilst no acute emotional trigger was identified in the medical history, it was noted the significant accumulating burden in managing her comorbid health conditions and the subsequent debilitating impact on her daily activities.

Under active monitoring and without active treatment, symptomatically, the patient recovered well and returned to her functional baseline. A subsequent echocardiogram 10 weeks after initial presentation (Video 3) showed recovery of systolic function, with significant improvement of LV from severely impaired (EF 35%) to good dynamic function (EF 60%–65%). Conversely to earlier echocardiogram, no obvious regional wall motion abnormality in the LV was seen.

## Discussion

Takotsubo cardiomyopathy is an under-recognized cardiovascular syndrome, with inconclusive underlying pathophysiology. The predominant theory is of circulating high levels of catecholamines, resulting in myocardial microvascular alterations leading to negative inotropy and resultant left ventricular contractile dysfunction.^3^ Newer evidence suggests systemic inflammatory activation, with associated pre-existing chronic inflammatory disorders, as seen in this patient. Takotsubo cardiomyopathy has been observed in a patient with underlying Hashimoto thyroiditis, rheumatoid arthritis, as well as pernicious anaemia.^6^ In fact, the presence of TCM with thyrotoxicosis has been predominantly linked to autoimmune Graves’ disease^7^; whilst originally believed to be due to thyrotoxicity, the predominance of Graves’ disease with TCM suggests a predisposition to autoimmune pathologies may heighten one’s risk of TCM. Furthermore, post-mortem examinations of hearts of human subjects who died during the acute phase of the condition demonstrated that macrophage infiltration is predominantly of the M1 pro-inflammatory type as opposed to the reparative M2 type.^8^ This hints at a mechanism where a predisposition to chronic inflammatory conditions exists in such patients, with acute stress encounters serving as the trigger for an enhanced systemic and hence myocardial inflammation. This highlights inflammatory activation as causative as opposed to consequential to TCM, given its greater propensity for development of the disease in low-grade chronic inflammatory states.

To this extent, whilst TCM has not been recognized as a potential cardiac complication of systemic diseases, there is evidence for the majority of patients experiencing a trigger of systemic disease flare-ups inducing psychological or physical stress prior to onset, rather than an emotional-type trigger. Even with flare-ups causing a catecholamine discharge, excess catecholamine may also induce a myocardial inflammatory response.^9^ Takotsubo cardiomyopathy occurring in the context of systemic lupus, vasculitis, rheumatoid arthritis, and Sjogren’s syndrome, as seen in the patient discussed, has recently been reported,^10^ suggesting common pathophysiological mechanisms uncovering underlying inflammatory processes, or a causal link such as relapse of the systemic disease.

Given the coincidence of TCM with autoimmune diseases, the case contributes to a wider discussion regarding the significance of considering TCM as a differential diagnosis of acute coronary syndrome (ACS) if no obstructive coronary artery disease is noted, especially given strong autoimmune and inflammatory comorbidities in the medical history. Early diagnosis is crucial in the acute phase, given its high rates of morbidity and mortality, with a 20% risk of congestive heart failure and 9% risk of ventricular arrhythmias of TCM patients, with complications of left ventricular wall rupture, thrombosis, and even cardiogenic shock reported.^11^ However, it remains clinically indistinguishable and resembles ACS in its clinical symptoms, ECG alterations and changes in standard laboratory parameters. The investigation with greatest diagnostic value is limited to coronary angiography and echocardiography; therefore, identification of patients presenting with suspicion of TCM and hence effective triage to urgent coronary imaging is crucial. Development of sensitive and selective biomarkers in combination with clinical scoring systems, such as the Inter TAK Diagnostic Score,^12^ may aid in more accurate triage and early diagnosis. Given the proposed immune-mediated pathophysiology, this has implications for the potential identification of immune-linked biomarkers, and the integration of inflammatory and autoimmune comorbidities in risk stratification scores.

In practice, the growing evidence highlights the importance of considering the diagnosis of TCM in patients known underlying chronic inflammatory and autoimmune diseases. It is also known that the disease has a strong female predominance, with a reported female-to-male ratio of around 5.3 to 1.^13^ The occurrence in predominantly women of postmenopausal ages, and the challenges of underdiagnosis, may reflect broader trends towards misinterpretation or dismissal of symptoms, overlooked in clinical practice in this patient cohort. Similarly, TCM is characterized by unique clinical characteristics with morphological variants, and incurs a substantial risk for adverse outcomes beyond the transient period, including ongoing chest pain, dyspnoea, or fatigue even after months of the acute event.^14^ The heterogeneous appearance of TCM hence needs to be recognized across medical disciplines, to maximize early identification and improvement of outcomes.

An alternative diagnosis here would be of Kounis syndrome, characterized by cardiovascular symptoms that occur secondary to allergic or hypersensitivity reactions on administration of a medical agent.^15^ Whilst this would have been fitting considering her recent drug history, as well as a history of severe hyperreactivity (Stevens–Johnson syndrome), the presence of a left ventricular aneurysmal appearance, caused by the akinesia of the apex and apical segments, supported TCM as a more likely diagnosis. Alternatively, whilst other types of myocardial infarction with non-obstructive coronary artery disease or spontaneous coronary artery dissection must be considered, the characteristic ballooning of the LV is pathognomonic for TCM.

Overall, a potential mechanism for TCM is a heightened susceptibility in patients with predisposition to chronic inflammatory conditions, with acute stress encounters serving as the trigger for an enhanced systemic and hence myocardial inflammation. This presents a novel immune-mediated component of TCM pathogenesis, which warrants further clinical and mechanistic investigation, with implications for disease-modifying treatments and recurrence prophylaxis. Additionally, given the challenges with diagnostic accuracy, this may highlight an important differential for acute myocardial infarction, to consider in patients with known underlying chronic inflammatory and autoimmune diseases.

## Supplementary Material

ytaf193_Supplementary_Data

## Data Availability

The images and findings analysed in this report are available from the corresponding author on reasonable request.

## Lead author biography

Yuqing Chen is a clinical medical student, studying at the University of Cambridge, conducting clinical placements in Addenbrooke's Hospital NHS Foundation Trust and at the East and North Hertfordshire NHS Trust. She completed her intercalation project (B.A.) at the Paulsen Lab at the Department of Physiology, Development, and Neuroscience, for which she was awarded a 1st Class Degree. She has previously been selected to conduct research at the Karolinska Institute as an Amgen Scholar, at the University of Tokyo, and has presented her work nationally and internationally.

## Notes

**Consent:** The authors certify that informed patient consent has been obtained for their images and other clinical information to be reported. Consent for publication has been obtained, in line with the COPE best practice guidelines, and that the individual(s) who is being reported on is aware of the possible consequences of that reporting.

**Funding:** Nil.
